# Potential Impact of the COVID-19 Pandemic on Public Perception of Water Pipes on Reddit: Observational Study

**DOI:** 10.2196/40913

**Published:** 2023-04-20

**Authors:** Zihe Zheng, Zidian Xie, Maciej Goniewicz, Irfan Rahman, Dongmei Li

**Affiliations:** 1 Goergen Institute for Data Science University of Rochester Rochester, NY United States; 2 Department of Clinical and Translational Research University of Rochester Medical Center Rochester, NY United States; 3 Department of Health Behavior Roswell Park Comprehensive Cancer Center Buffalo, NY United States; 4 Department of Environmental Medicine University of Rochester Medical Center Rochester, NY United States

**Keywords:** water pipes, Reddit, COVID-19, COVID-19 pandemic, public perception

## Abstract

**Background:**

Socializing is one of the main motivations for water pipe smoking. Restrictions on social gatherings during the COVID-19 pandemic might have influenced water pipe smokers’ behaviors. As one of the most popular social media platforms, Reddit has been used to study public opinions and user experiences.

**Objective:**

In this study, we aimed to examine the influence of the COVID-19 pandemic on public perception and discussion of water pipe tobacco smoking using Reddit data.

**Methods:**

We collected Reddit posts between December 1, 2018, and June 30, 2021, from a Reddit archive (PushShift) using keywords such as “waterpipe,” “hookah,” and “shisha.” We examined the temporal trend in Reddit posts mentioning water pipes and different locations (such as homes and lounges or bars). The temporal trend was further tested using interrupted time series analysis. Sentiment analysis was performed to study the change in sentiment of water pipe–related posts before and during the pandemic. Topic modeling using latent Dirichlet allocation (LDA) was used to examine major topics discussed in water pipe–related posts before and during the pandemic.

**Results:**

A total of 45,765 nonpromotion water pipe–related Reddit posts were collected and used for data analysis. We found that the weekly number of Reddit posts mentioning water pipes significantly increased at the beginning of the COVID-19 pandemic (*P*<.001), and gradually decreased afterward (*P*<.001). In contrast, Reddit posts mentioning water pipes and lounges or bars showed an opposite trend. Compared to the period before the COVID-19 pandemic, the average number of Reddit posts mentioning lounges or bars was lower at the beginning of the pandemic but gradually increased afterward, while the average number of Reddit posts mentioning the word “home” remained similar during the COVID-19 pandemic (*P*=.29). While water pipe–related posts with a positive sentiment were dominant (12,526/21,182, 59.14% before the pandemic; 14,686/24,583, 59.74% after the pandemic), there was no change in the proportion of water pipe–related posts with different sentiments before and during the pandemic (*P*=.19, *P*=.26, and *P*=.65 for positive, negative, and neutral posts, respectively). Most topics related to water pipes on Reddit were similar before and during the pandemic. There were more discussions about the opening and closing of hookah lounges or bars during the pandemic.

**Conclusions:**

This study provides a first evaluation of the possible impact of the COVID-19 pandemic on public perceptions of and discussions about water pipes on Reddit.

## Introduction

Water pipe tobacco, also known as “hookah,” is a combustible tobacco product usually used in a group setting [1]. A previous systematic review found that the major motivations for water pipe smoking are socialization, relaxation, pleasure, and entertainment [2]. Water pipe tobacco smoking (WTS) often involves the use of an apparatus that heats the tobacco and passes the smoke through water before it can be inhaled through a hose by the user. Data from wave 1 to 3 of the Population Assessment of Tobacco and Health (PATH) study showed that young adults (aged 18-24 years) had a higher prevalence of WTS than youth and adults aged 25 years or older [1]. The prevalence of past-30-day WTS in young adults was 9.2%, compared to 0.7% in youth and 1.2% in adults aged 25 years or older, according to PATH wave 3 data collected between 2015 and 2016 [1]. A previous online survey study of US adults aged 18 to 30 years found that positive attitudes and perceived peer acceptability of WTS were significantly associated with WTS in young adults [3].

Similar to many other tobacco products, WTS is related to many health issues, including lung cancer, respiratory illness, low birth weight, and periodontal disease, as well as bronchitis, metabolic syndrome, cardiovascular disease, and mental health [4,5]. Many water pipe users prefer flavored tobacco [6]. The most popular flavors are fruit flavors, followed by sweets, spice, alcohol, and other beverages [6]. It has been reported that the main motivations for WTS are socialization, relaxation, pleasure, and entertainment [2]. Homes and hookah lounges are the most common places where people use water pipe tobacco [7].

It has been shown that smoking behaviors were affected by the recent COVID-19 pandemic [8]. COVID-19 is an infectious disease caused by SARS-CoV-2 [9]. The first case of COVID-19 was diagnosed in December 2019 in Wuhan, China, and it was declared a global pandemic on March 11, 2020, by the World Health Organization (WHO) [10]. To mitigate the spread of the disease, many countries, including the United States, India, and China, enforced lockdowns on the economy and cities [11,12]. Studies have shown many positive outcomes of lockdowns, such as a significant decrease in the growth rate of confirmed cases, as well as improved global air quality and lower pollution [11,13]. On the other hand, the lockdowns caused mental health problems, such as anxiety, depression, loneliness, sleep difficulties, and hyperarousal, as well as a higher tendency to overeat and experience obesity [14-16]. Some studies showed that psychiatric emergency admissions increased during the lockdowns, raising a debate on how the pandemic might have affected mental health [17,18]. The COVID-19 pandemic and the accompanying lockdown policies have been proven to have influenced tobacco smoking [19,20]. Smoking prevalence was shown to have decreased in urban counties in the United States [20]. In addition, the vaping rate among youth and young adults declined during the pandemic in the United States [19]. A recent online survey study conducted among 1223 US adults in 2020 showed that a more severe perception of smoking-related COVID-19 risks was associated with a higher likelihood of smoking reduction and quit attempts [21]. A cross-sectional household survey study conducted in England in 2020 showed that a minority of e-cigarette users attempted to quit vaping because of COVID-19 [22]. Another survey study of Israeli smokers showed increases in both the number of smokers and attempts to quit smoking due to COVID-19 [23]. Given water pipes are often smoked in groups during social gatherings, it is possible that the lockdowns during the COVID-19 pandemic affected water pipe use.

As of January 2021, Reddit had more than 50 million daily active users, 100,000 active communities, and 13 billion posts and comments [24]. Reddit has been widely used for discussing many public health events [25]. Due to its increasing popularity, Reddit has been used to study public perceptions and discussions of tobacco products. For example, to understand reasons why people with mental health problems smoke e-cigarettes, a group of scientists analyzed 3263 posts on Reddit and found that the main reasons for e-cigarette use included self-medication, freedom and control, and motivation from caregivers and online communities [26]. Several studies have used Reddit data to study public perceptions of flavored e-cigarette use and related health symptoms [27-29]. Another study analyzed Reddit posts related to oral nicotine pouches and found that people generally had a positive attitude toward oral nicotine pouches [30]. Since the pandemic started, active discussions on Reddit have made it a useful resource to study the influence of the COVID-19 pandemic. Recently, Reddit has been used to find patterns of posts that imply mental health problems and identify at-risk users on the platform during the COVID-19 pandemic [31].

In this study, we aimed to understand how the COVID-19 pandemic influenced public perceptions and discussions of water pipe tobacco smoking on Reddit through interrupted time series (ITS) data analyses, sentiment analyses, and topic modeling. More importantly, we aimed to investigate whether the COVID-19 pandemic had an impact on water pipe smoking behaviors, such as reducing use in hookah lounges or bars during the pandemic, given water pipes are commonly smoked during social events. Our study provides an initial but important evaluation of the potential impact of the COVID-19 pandemic on water pipe perception and discussion, as well as potential water pipe behavior changes, through social media data mining.

## Methods

### Data Collection and Preprocessing

Reddit posts (comments) from December 1, 2018, to June 30, 2021, were downloaded from a Reddit archive (PushShift). We extracted posts related to water pipes using a set of keywords from a previous study [32], including *water pipe*, *hookah*, *shisha*, *narghile*, *argileh*, *hubble-bubble*, *goza*, *borry*, *qaylan*, *mada’a*, *mouassal*, *jurak*, *tumbak*, *hooka*, *sheesha*, and *hubblebubble*. In total, 62,699 water pipe–related Reddit posts were obtained.

A multi-filter process was used to preprocess the Reddit data. First, we applied a filter to obtain all the posts written in English. Second, we applied additional filters to ensure that all posts were related to water pipes. For example, Reddit posts that contained “shisha octane,” “waterpipe shotgun,” and “hookah attack” were discussing games like *Rust* or *Rainbow Six Siege* instead of water pipe smoking. To eliminate such noise, we removed Reddit posts that contained the above combinations of keywords and Reddit posts from game subreddits, including /ps4, /xbox, /playrust, /rainbow6, /boombeach, /siegeacademy, /r6proleague, and /valorant. Third, commercial Reddit posts were removed if they contained keywords such as *discount*, *deal*, and *dealer*, or if their usernames included keywords such as *dealer*, *water pipe*, or *hookah*.

### Location Analysis

To determine whether the COVID-19 pandemic affected water pipe smoking behavior due to restrictions on social gatherings, we conducted a location analysis. We constructed a location data set for Reddit posts that mentioned specific locations according to the most common locations of water pipe smoking mentioned in the posts. First, we performed an item count that included single words, bigrams, and trigrams. Then, we manually examined the items with high frequency to identify location-related items. We classified these items into 2 main categories: *home* and *lounge/bar*. The *home* category included *home*, *house*, *living room*, and *dining room* while the *lounge/bar* category included *lounge*, *lounges*, *bar*, *bars*, *cafe*, *cafes*, *coffee shop*, *coffee shops*, *strip club*, and *strip clubs*. The location data set consisted of 9344 Reddit posts mentioning either *home* or *lounge/bar*.

### Temporal and ITS Analysis

To study trends in the discussion of water pipe tobacco on Reddit, we calculated the number of water pipe–related Reddit posts per week, as well as the number of Reddit posts that mentioned either *home* or *lounge/bar* per week. An ITS analysis was used to determine if trends before the COVID-19 pandemic were different from trends during the COVID-19 pandemic, for either *home* or *lounge/bar*. In an ITS analysis, time series are segmented by the intervention point and segmented regression is used to evaluate the changes in level and slope before and after the intervention point [33]. In our study, we set the intervention point as March 11, 2020, which is the day that the WHO declared COVID-19 to be a global pandemic [10]. We identified 155 posts that were comments on a single popular post made on February 19, 2020, about a husband smoking water pipe tobacco while his wife was pregnant. After carefully examining these posts, we manually removed all of them, since they were not related to WTS. The ITS analyses were conducted using SAS (version 9.4; SAS Institute). The significance level of the test was set at 5% for 2-sided tests.

### Sentiment Analysis

We generated a sentiment score for each post using VADER (Valence Aware Dictionary and Sentiment Reasoner). VADER is designed for qualitative sentiment analysis of social media using a list of lexical features combined with rules about conventions for expressing emotions [34]. A post is considered to have a positive attitude if it has a score equal to or higher than 0.05, a negative attitude if the score is equal to or lower than –0.05, and a neutral attitude if the score is between –0.05 and 0.05. To determine if there was any change in the proportion of posts with different sentiments before and during the COVID-19 pandemic, we performed a 2-proportion *z* test with a significance level of .05.

### Topic Modeling

To identify and compare the topics discussing water pipes on Reddit, we performed topic modeling using the latent Dirichlet allocation (LDA) model on posts made before and during the COVID-19 pandemic. The LDA model is a generative statistical model that can be used to find topics in documents [35]. The algorithm first calculates the probabilities of each word appearing in each topic and then defines each topic with the words that have the highest possibility of appearing in that topic. We chose the optimal number of topics based on the maximum coherence score. Sentences from the posts were transformed to lowercase letters, and stop words, such as *the*, *am*, and *you*, were removed using the Natural Language Toolkit (NLTK Team) in Python. Additionally, the words were lemmatized using *spacy* (ExplosionAI GmbH) [36] in Python.

### Ethics Approval

Only publicly available Reddit posts were used for this study. There was no identifying information on Reddit users in this study. To protect human subjects included in this study, this study was reviewed and approved by the Research Subjects Review Board of the Office for Human Subject Protection at the University of Rochester (STUDY00006570).

## Results

### Discussion of Water Pipes on Reddit

**Figure 1:**
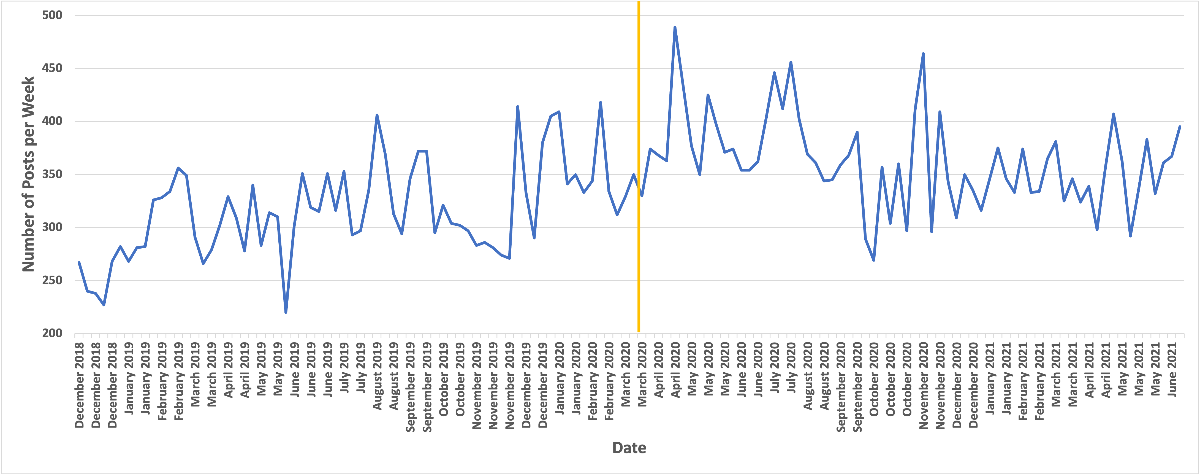
Longitudinal trend in Reddit posts related to water pipes.

From 62,699 Reddit posts extracted from the Reddit archive based on water pipe–related keywords, we identified 56,462 English Reddit posts, among which 51,387 were related to water pipes. Further removal of promotion posts resulted in 45,765 Reddit posts related to water pipes, which were used for further analysis. To understand trends in the discussion of water pipes on Reddit over time, we examined the number of posts related to water pipe tobacco per week from December 1, 2018, to June 30, 2021 (Figure 1). The vertical line marks March 11, 2020, the starting date of the COVID-19 pandemic. As shown in Figure 1, ITS analysis showed that the discussion of water pipe tobacco on Reddit was significantly increasing before the COVID-19 pandemic (*P*<.001). After the announcement of the COVID-19 pandemic, the popularity of water pipe–related Reddit posts significantly decreased (*P*<.001). ITS analysis further showed that the average number of water pipe–related posts per day during the pandemic was significantly higher than before the pandemic (*P*<.001).

**Figure 2:**
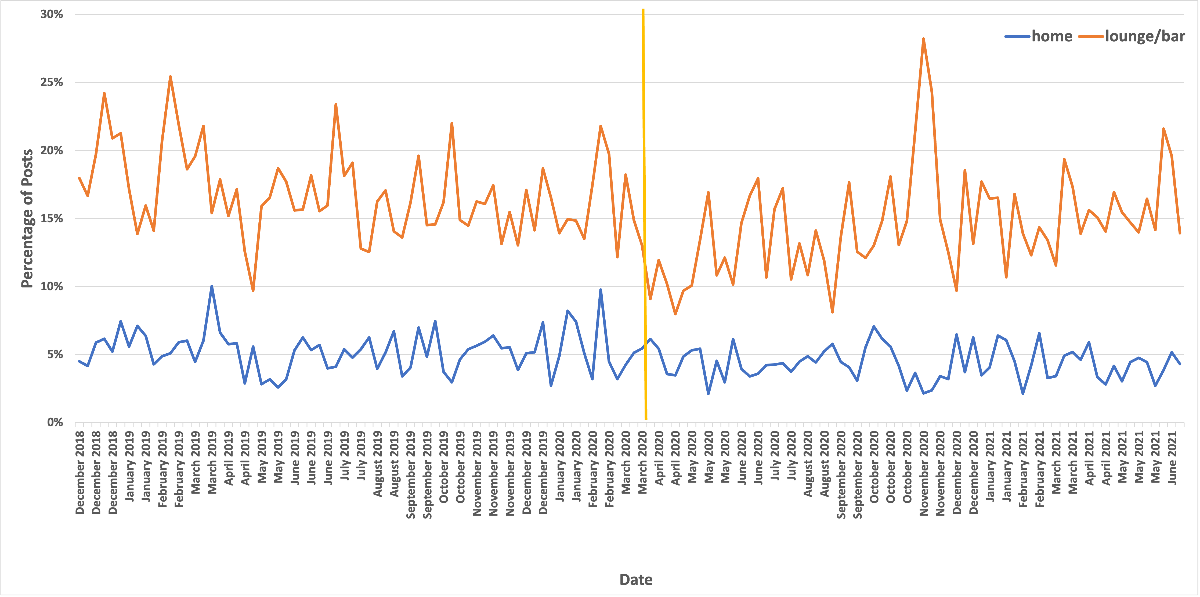
Longitudinal trend in the proportion of water pipe–related Reddit posts mentioning either *home* or *lounge/bar*.

To examine whether the pandemic had any impact on the location of water pipe tobacco use, we first identified posts mentioning the 2 most common locations for water pipe use, homes and lounges or bars (Figure 2). In total, we identified 2194 posts mentioning *home/house*, and 7150 posts mentioning *lounge/bar*. Further ITS analysis showed that discussion of smoking water pipe tobacco at home remained stable over the period of study (*P*=.29). In contrast, discussion about smoking water pipes at lounges or bars significantly decreased in the time leading up to the pandemic (*P*=.004), then significantly increased after the announcement of the pandemic (*P*<.001). Compared to before the pandemic, the average number of posts mentioning smoking water pipes at lounges or bars was significantly lower during the pandemic (*P*<.001). In addition, we identified a peak in November 2020 that resulted from 70 posts related to a news story about closing hookah lounges in Saskatoon, Saskatchewan.

### Sentiment Changes in Water Pipe–Related Posts Before and During the COVID-19 Pandemic

To understand whether the COVID-19 pandemic had any impact on the sentiments of water pipe–related posts, we performed a sentiment analysis of water pipe–related posts before and during the COVID-19 pandemic. Before the pandemic, 59.14% (12,526/21,182) of posts had a positive attitude, 21.44% (4541/21,182) of posts had a negative attitude, and 19.43% (4115/21,182) of posts had a neutral attitude. After the pandemic, 59.74% (14,686/24,583) of posts had a positive attitude, 21.01% (5164/24,583) of posts had a negative attitude, and 19.25% (4733/24,583) of posts had a neutral attitude. Further statistical analysis using 2-proportion *z* tests showed that there was no significant change in the proportion of posts with positive attitude (*P*=.19), negative attitude (*P*=.26), or neutral attitude (*P*=.65) before and after the pandemic.

### Topics Discussed in Water Pipe–Related Reddit Posts

LDA topic modeling was used to identify popular topics related to water pipes on Reddit before and during the pandemic. As shown in Table 1, water pipe–related posts (n=21,182) had 6 major topics before the pandemic, including “friends spending time together” (3220, 15.2%), “hookah culture in different countries” (2284, 10.78%), “discussion about waterpipe accessories” (3064, 14.46%), “getting bad feelings when using waterpipe tobacco with other substances” (3755, 17.73%), “smoking hookah at hookah bars/lounges” (4114, 19.42%), and “discussion about coal and shisha flavor” (4745, 22.4%). The 6 most popular topics in Reddit posts after the pandemic started (n=24,583; Table 2) included “friends spending time together” (3453, 14.05%), “getting bad feelings when using waterpipe tobacco with other substances” (3651, 14.85%), “discussion about coal and shisha flavor” (3662, 14.9%), “opening and closing hookah bars/lounges” (4430, 18.02%), “discussion about waterpipe accessories” (4841, 19.69%), and “good feelings about hookah” (4546, 18.49%). The keywords and associated example Reddit posts are also included in both Tables 1 and 2.

**Table 1:** Topics discussed in water pipe–related Reddit posts (n=21,182) before the pandemic.

Topic	Percentage of tokens, n (%)	Keywords	Example quotations
Friends spending time together	3220 (15.2)	friend, time, back, guy, start, day, feel, leave, year, end	“Looking through this thread and seems to be like I’m in the minority. I do, and I do it a lot. Granted, only with my close friends, but I have quite a few of those. We call them “heart to hearts”, and they are usually accompanied by whiskey, hookah, and petting my too-needy-but-cute-as-shit pittie.” “I took the truck back after he was done camping one night and went to the hookah bar with a friend and to the location and back windows were wide open and we had our shirts over our mouths as we were yelling in fear the entire drive...”
Hookah culture in different countries	2284 (10.78)	people, man, thing, give, post, life, find, world, show, country	“In Bosnia, hookah is known as Shisha. A lot of Bosnians have moved to Germany, more than any other European country, so maybe that’s why.” “I’m from the middle east. Our culture includes: Hookah, Thobes.... Making houses out of fur (usually for camping). The shit stuff you’re thinking about is islam.”
Discussion about waterpipe accessories	3064 (14.46)	water, make, work, put, pipe, long, hit, water pipe, bit, bong	“Solo 2 is so good... get the 14mm water pipe adaptor for it and it’s a vong beast!” “When washing your bowl (if you’re like me who smokes 100 flavor on same bowl and same hookah). Fully wash the bowl with hot water, use normal towel to dry it, and place it on stove to fully dry it.”
Getting bad feelings when using waterpipe tobacco with other substances	3755 (17.73)	smoke, hookah, tobacco, cigarette, smoking, vape, bad, weed, time, nicotine	“Almost sitting *[sic]* cigarettes, down to 1 or 2 a day from 7-8...by next week it should be once in 2 days or less. But I don’t smoke a cig today and did sheesha and went to the gym straight. IT WAS HORRIBLE…Never again. And not even going to do sheesha ever.” “My girlfriend made me try weed before I tried LSD, even though I had no interest in weed at all. I really don’t like weed at all. It feels like it clouds my mind, and I feel like I am too heavy…I have smoked hookah for years, so when I went to smoke weed, I kept clearing the bowl in one hit... Apparently that isn’t good to do for a first timer.”
Smoking hookah at hookah bars/lounges	4114 (19.42)	bar, hookah, good, lounge, place, lot, hooka, great, love, pretty	“Hound dogs pizza! If you’re a smoker hookah bars are usually open really late. Also diners are a good option, fitzys on shrock rd is 24 hours and waffle house and steak and shake.” “More than Fumari and Starbuzz I see. I wonder if it is allowed to be smoked at the hookah lounges in US.”
Discussion about coal and shisha flavor	4745 (22.4)	hookah, bowl, good, shisha, coal, buy, hose, flavor, clean, brand	“Sorry, never personally tried Starbuzz Carbine, cannot really compare the draw differences between them...” “Serbetli amazing shisha, strong flavor and thick clouds!”

**Table 2:** Topics discussed in water pipe–related Reddit posts (n=24,583) during the pandemic.

Topic	Percentage of tokens, n (%)	Keywords	Example quotations
Friends spending time together	3453 (14.05)	friend, people, guy, talk, play, back, life, call, time, show	“Before I moved here from Omaha I used to frequent the hookah lounge all the time!... My boyfriend and I were thinking about going to one of them the other night but didn’t know which one was gonna be the best option at the time. I’ll definitely have to check out both though!” “This was just a few weeks ago. Met a girl through hanging with mutual friends at a hookah bar. We spent a couple hours talking to each other, and she asked our friend to give her my number...”
Getting bad feelings when using waterpipe tobacco with other substances	3651 (14.85)	smoke, time, people, feel, day, year, smoking, bad, cigarette, weed	“I think it can be done. I do hookah sometimes. That doesn’t mean I’m ever smoking again! I even, can’t stand the smell of cigarettes anymore. In your experience and your strength, there are no rules!” “Smoke a cigarette or cigar or hookah or vape-tobacco, marijuana, herbal, or ANYTHING!! YUCK! Smelly! Stinky! Obnoxious! And Cancer!... did I mention lung cancer??”
Discussion about coal and shisha flavor	3662 (14.9)	tobacco, bowl, smoke, hookah, coal, shisha, flavor, water, taste, heat	“Anything by Mason Shishaware. The Gravyl bowl is the best hookah bowl I’ve ever smoked hands down.” “It depends on what do you like ( I like mixing dark leaf with white, using neutral flavors of dark leaf (dark side tobacco ( as base (grapefruit, orange, banana ( and add strong tastes of daily hookah (exotic fruits, different cocktails, mint, Red Bull)”
Opening and closing hookah bars/lounges	4430 (18.02)	bar, lounge, place, night, open, home, back, sit, room, close	“til not all non-essential businesses were told to close my distant cousins hookah cafe in a city centre in jabodetabek is apparently still open” “I really miss Hookah since I usually go out to those shops to smoke em. How much would it cost to buy home a hookah set?”
Discussion about waterpipe accessories	4841 (19.69)	Hookah, hose, buy, pipe, make, glass, clean, quality, base	“By whip, do you mean like a hookah hose? If so, I LOVE concentrates through one. ZC Glass makes some sweet parts if you ever need splitters or new mouthpieces.” “Glass Lung sells hookah style mason jar bongs that have 2 14 mm female adapters so you can attach a whip and a bowl to. They’re pretty cool.”
Good feelings about hookah	4546 (18.49)	Hookah, good, work, love, pretty, great, lol, hit, hooka, big	“Wow, I love the way this hookah looks. Especially the ‘vase’! Hot damn I want that!” “That’s pretty awesome! I have a Starbuzz Carbine that has the 360 degree swivel...very nice feature!”

## Discussion

### Principal Findings

In this study, we showed that the discussion of water pipes on Reddit was gradually increasing until the beginning of the COVID-19 pandemic, and then gradually decreased during the pandemic. There was more discussion about water pipes on Reddit during the pandemic than before the pandemic. While the proportion of posts mentioning water pipe use at home did not change during the study period, the proportion of posts mentioning water pipe use at lounges or bars significantly decreased at the beginning of the pandemic, and gradually increased. Positive water pipe–related posts were dominant, and this did not change with the COVID-19 pandemic. The discussion on water pipes was similar before and during the pandemic. There was more discussion about the opening and closing of hookah bars and lounges during the pandemic.

By examining trends in all water pipe–related posts on Reddit and performing an ITS analysis, we showed that the number of posts related to water pipe tobacco had a growing trend before the pandemic. The announcement of COVID-19 as a global pandemic had a positive effect on this increasing trend, which might be due to the discussion of pandemic lockdowns. After the sharp increase at the beginning of the pandemic, the number of related posts started to decrease.

### Comparison With Prior Work

Our findings are aligned with those of a study on smoking in Saudi Arabia that concluded that the use of cigarettes and water pipe tobacco has slightly decreased while e-cigarette use has significantly increased since the pandemic [37]. Our study focused on noncommercial water pipe smoking–related Reddit posts. It would be interesting to see how the sales of water pipe tobacco and accessories changed during the pandemic; this was beyond the scope of this study and will be explored in the future.

In this study, we used location-specific keywords to explore the impact of the COVID-19 pandemic on mentions of different locations (hookah bars/lounges or home). This may be the first study to use social media data to examine how the pandemic affected water pipe use. Our temporal analysis showed that the proportion of discussions about hookah lounges and bars significantly decreased at the beginning of the pandemic and slowly increased during the pandemic. This trend aligns with the timeline for the pandemic: major lockdowns started in March 2020, when the state of California issued a stay-at-home order; then, in May 2020, the US Centers for Disease Control and Prevention released guidance for reopening the country, followed by the gradual reopening of the economy in the United States [38]. The number of people under confinement worldwide reached its highest point on April 5, 2020, and then started to decrease [39]. Therefore, our results suggest that the pandemic might have had some potential impact on water pipe use, based on mentions of different locations (such as bars and lounges) on Reddit during the pandemic. Due to the lockdown at the beginning of the pandemic, many hookah bars or lounges were closed or not accessible, which might have led to fewer mentions of hookah bars and lounges on Reddit. With fewer restrictions during the later lockdowns, some hookah bars and lounges started to open, and people began searching for possible social activities, such as hanging out at hookah bars and lounges with their friends, which might have resulted in more mentions of hookah bars and lounges on Reddit. The proportion of discussions about using water pipe tobacco at home was low, which indicates that fewer people smoked water pipe tobacco at home in general.

Most of the Reddit posts related to water pipes had positive sentiments in our study. Sentiment analysis of water pipe–related posts on Twitter showed that 59.5% (352,116/591,792) of tweets had a positive attitude, while 30% (177,537/591,792) had a negative attitude, and 10.5% (62,139/591,792) had a neutral attitude [40]. We further showed that there was no change in the sentiment of water pipe–related posts before and during the pandemic, suggesting that the pandemic did not impact the public perception of water pipes.

By comparison, we showed that the most popular topics in water pipe–related posts were similar before and during the pandemic, including “friends spending time together while smoking waterpipe tobacco,” discussions about waterpipe-related products,” and “getting bad feelings when using waterpipe tobacco with other substances like cigarettes and weed.” However, we did notice that while discussion of hookah bars and lounges was present in posts both before and during the pandemic, the focus of the discussion shifted to the opening and closing of hookah bars and lounges after the pandemic started. In addition, before the pandemic people frequently posted and discussed hookah culture around the world, and this became less popular during the pandemic. The great number of travel bans and restrictions caused by the pandemic might be one of the possible reasons for this change [41].

### Limitations

There are several limitations to our study. First, as the majority of Reddit users are from North America, our data set may not be representative of the global discussion about water pipes during the pandemic [25]. Given the unique social context of water pipe smoking in the United States, the findings are not generalizable to all countries, especially those countries where water pipe smoking may primarily occur alone or in private homes [42]. Due to the lack of detailed geolocation information for Reddit users, we could not compare the potential impact of the COVID-19 pandemic on water pipe tobacco smoking in Canada and the United States. Therefore, it will be important in the future to examine how the pandemic might affect the use of water pipe or other tobacco products in different countries with different lockdown policies, especially how tobacco product users changed their user behaviors. Second, the Reddit data that we used in this study were historical data, so some water pipe–related Reddit posts might have been deleted, which would have introduced bias to our results. Third, although we examined temporal trends in the percentage of discussions about water pipes and specific locations, we could not distinguish posts about actually smoking water pipes in hookah bars and lounges from discussions about reopening hookah bars and lounges. Fourth, some water pipe users who searched for other locations during the pandemic might not have mentioned it on Reddit, which could also have brought bias into our results. Finally, the sentiment analysis was performed at the post level, so it may not have reflected the actual attitude of Reddit users toward water pipes.

### Conclusions

Our study provides a thorough analysis of the potential influence of the COVID-19 pandemic on public perceptions and discussions about water pipes on Reddit by performing temporal analysis and comparing sentiments and topics discussed before and during the pandemic. Our findings show that during the pandemic, especially during lockdowns, mentions of opening or closing of hookah bars and lounges on Reddit gradually increased, suggesting that people were searching for collaborative activities, such as water pipe tobacco smoking in hookah bars and lounges with their friends. Our study shows the potential impact of the pandemic on water pipe tobacco smoking, such as the closing of hookah bars and lounges; this might create an opportunity for public health authorities to communicate with the public during lockdowns about what kind of health collaborative activities they should search for instead of water pipe tobacco smoking. Our study also provides another valid data source obtained from social media for studying the pandemic and any other important public health issues, considering the increasing prevalence of social media use in the modern world.

## Abbreviations

FDA: Food and Drug Administration
ITS: interrupted time series
LDA: latent Dirichlet allocation
NIH: National Institutes of Health
VADER: Valence Aware Dictionary and Sentiment Reasoner
WTS: water pipe tobacco smoking

## Data Availability

The data sets and scripts generated during and/or analyzed during the current study are available on request from the corresponding author (DL).
